# A review of the changes to the licensing of influenza vaccines in Europe

**DOI:** 10.1111/irv.12351

**Published:** 2015-12-11

**Authors:** Leonoor Wijnans, Bettie Voordouw

**Affiliations:** ^1^ Medicines Evaluation BoardUtrechtThe Netherlands

**Keywords:** influenza, pandemic vaccine, regulatory guidance, seasonal vaccine, strain change, zoonotic vaccine

## Abstract

In 2014, the European Committee for Medicinal Products for Human Use (CHMP) published a draft regulatory guideline for the evaluation of influenza vaccines. Following a public consultation round, the final guidance will be published in the near future. Here, we highlight the main changes in the clinical section in this guideline and discuss the background to these changes and whether the new consolidated guidance document can be expected to achieve a better understanding of the performance of seasonal, zoonotic and pandemic influenza vaccines during the regulatory licensing process. The new influenza guideline reflects a changed approach to the regulatory assessment of influenza vaccines, resulting in the abolition of serological criteria, known as the CHMP criteria, which have been the mainstay for evaluating the influenza vaccine immunogenicity for several decades. The new guideline adopts a more diversified approach to the measurement and reporting of the immune response to influenza vaccines and sets a requirement to conduct clinical outcome trials in young children. Importantly, more emphasis is placed on the post‐licensure monitoring of the benefit risk of influenza vaccines, including a request for continuous monitoring of efficacy and enhanced safety surveillance. Despite the improvements these new requirements will expectedly bring to the regulatory assessment of influenza vaccines, major challenges remain which cannot be overcome by new guidance alone. Ongoing initiatives in which academia, manufacturers, public health institutes and regulators work together to address these challenges are central to the development of robust tools to evaluate and monitor performance of influenza vaccines in the future.

## Introduction

In 2014, the European Committee for Medicinal Products for Human Use (CHMP) published a draft regulatory guideline for the evaluation of influenza vaccines.^1^ This guideline is intended to update the multitude of guidance documents in Europe which cover quality, and non‐clinical and clinical regulatory requirements for seasonal, zoonotic or pandemic vaccines into a consolidated guidance document for the development of new influenza vaccines.

As outlined in a concept note published in 2011,^2^ the use of influenza vaccines is in certain aspects based upon “long‐standing practices rather than rigorous scientific appraisal”. The experience gained from the influenza A(H1N1)2009 pandemic brought into question the validity of several aspects of the existing regulatory guidelines. In particular, the assessment of the immune response, which focuses on haemagglutination inhibition (HI) and single radial haemolysis (SRH) assays, was considered to be in need of revision.^3^ Furthermore, a lack of understanding of the effect of vaccination on certain subpopulations, such as young children, called for improvements to existing guidelines.^3^, ^4^

In this article, we highlight the main changes in the clinical section of the new influenza guideline and their scientific background, and discuss whether this consolidated guidance document can be expected to achieve a better understanding of the performance of seasonal, zoonotic and pandemic influenza vaccines during the regulatory licensing process. We consider the evidence and current understanding surrounding the evaluation of the immune response, efficacy and safety of influenza vaccines and how the proposed guideline might improve the understanding on the effect of influenza vaccines on different subpopulations.

## European regulatory framework

In Europe, influenza vaccines are either licensed on a Europe‐wide scale where all Member States are involved, referred to as a ‘central procedure; via procedures in which selected Member States are involved, referred to as “decentralized procedure” or “mutual recognition procedure”; or on a national level. Whilst this provides a diverse regulatory landscape, in general all Member States adhere to scientific and regulatory guidance as set out by the CHMP. The CHMP is the committee of the European Medicines Agency (EMA), responsible for preparing the Agency's scientific opinion regarding the licensing of human medicinal products, including vaccines. Regulatory guidelines, such as the new influenza guideline, inform industry on the minimum requirements for licensing of new medicinal products. They reflect the information needed to determine the benefit risk balance of a product and to adequately describe the characteristics of the product to ensure safe and effective use. In the drafting of these guidelines, the CHMP is supported by several expert groups, such as the Vaccine Working Party and the Biologics Working Party. The Pharmacovigilance Risk Assessment Committee (PRAC) is the committee of the EMA responsible for the assessment and monitoring of safety issues that arise post‐licensure.

The newly revised influenza guideline distinguishes three types of influenza vaccines: those aimed at protecting individuals against seasonal, annually recurring influenza; zoonotic vaccines that contain an influenza virus strain of animal origin and which were previously referred to as pre‐pandemic vaccines; and pandemic influenza vaccines which are intended for use in a pandemic and which include pandemic preparedness vaccines, formerly referred to as pandemic mock‐up vaccines. The revised guideline integrates recommendations for new influenza vaccines; however, it clearly indicates that it does not intend to cover novel constructs, for example vaccines targeted at epitopes other than those on the haemagglutinin stalk.

## Serological correlates of protection: moving away from the existing paradigm for establishing efficacy of influenza vaccines

Traditionally, efficacy of inactivated influenza vaccines for regulatory assessment in Europe has been estimated through the determination of immunogenicity with serological assays. This assessment focused primarily on the HI assay for which seroprotection was defined as a cut‐off of HI ≥ 1:40, or the SRH assay for which a zone area of 25 mm^2^ is defined as a protective threshold. These cut‐offs stem from limited data from challenge studies conducted decades ago, demonstrating a relationship between HI titres and infection rates. These studies found that a pre‐challenge serum HI titre of 18–36^5^ measured by HI assays or 42–44^6^ measured by SRH assay correlated with 50% protection against infection. The serological response would be assessed by applying a set of criteria commonly referred to as the CHMP criteria (Table 1). For the annual variation of influenza strains in seasonal inactivated vaccines, one or more of the CHMP criteria had to be met. For pandemic vaccines, all three of the criteria had to be met.

**Table 1:** European CHMP criteria for evaluation of influenza vaccine immunogenicity

	Adults	Older adults (>60 years)
GMT increase	2·5	2
Seroconversion/significant increase^a^	40%	30%
Seroprotection^a^	70%	60%

a In HI tests, seroconversion corresponds to: negative pre‐vaccination serum (HI < 1:10), post‐vaccination serum HI ≥ 1:40; pre‐vaccination serum >1:10, significant increase: at least a fourfold increase in titre. Seroprotection corresponds to the percentage with serum HI ≥ 1:40. Alternative criteria have been defined for the SRH assay.

There has been a growing recognition that relying on a single serological cut‐off for determining the benefit of different influenza vaccines for different subgroups and different vaccine constructs is not the most informative approach^7^ and that the appropriateness of the defined correlate of HI ≥ 1:40 can be questioned.^3^, ^8^, ^9^, ^10^

Challenge studies on which the protective thresholds are based were performed in healthy adults with attenuated strains.^5^ However, influenza vaccines are intended not only to protect healthy adults but also to protect vulnerable children, older adults and adults with underlying comorbidities against consequences of natural infections with virulent influenza strains. Whether the correlates established in these challenge studies^5^ can be transferred to these situations has not been established. For example, one study identified that in children, an HI titre >1:110 would predict 50% of clinical protection and a titre of 1:330 would predict 80% of protection.^11^ A second study could not consistently predict protection with HI titres in healthy adults,^8^ and in older adults, it has been suggested that cell‐mediated immunity (CMI) rather than humoral immunity would be associated with protection.^12^ Serological assays are not an appropriate measure for the assessment of immunity against live attenuated influenza vaccines.^9^

Nonetheless, for decades the regulatory assessment of vaccines has relied on these criteria and correlates of protection even though their suitability to the situations for which they have been applied has not been established. The use of these correlates has arguably resulted in a loss of opportunity to gain knowledge and understanding of the functioning of influenza vaccines. Moreover, presenting and communicating study results against these criteria may have led to a false sense of security from the impression that a vaccine will convey a level of protection in the target population, when in fact this has not been established. The abolishing of these criteria marks a major shift in regulatory thinking and paves the way to a more evidence‐based approach for the assessment of vaccine performance.

A potentially more pressing problem arising from reliance on serological assays is the lack of standardization.^13^, ^14^ An international collaborative study which evaluated assay reproducibility for pandemic influenza H1N1 found the interlaboratory variation in the HI and virus neutralisation (VN) assay to be up to sixfold and sevenfold, respectively,^15^ whilst interlaboratory variation has been found up to 80‐fold for HI assays and 109‐fold for VN assays.^16^ This forms a clear impediment to reliance on these serological assays for the determination of efficacy. Comparisons of vaccine performance between different studies, including those performed in different seasons, cannot be made, limiting the accrual of understanding in the performance of different vaccines.

In response to these issues, the new guideline firstly requests a more diversified characterisation of the immune response and secondly the guideline no longer relies on serological assays with a predefined protective threshold to establish benefit.

## Requirements on immunogenicity

The guideline requests a more comprehensive package on the immunogenicity which includes – next to quantifying the HA antibody response – quantifying functional antibodies by determining neutralising antibody titres with VN assays and assessing the CMI in a subset of trial participants, in particular in older adults.

All these assays come with limitations. The VN assay is considered a suitable alternative to HA‐based assays^3^; however, the optimal protocol for this assay is yet to be identified.

Although the assessment of CMI is regarded as an integral part of the characterisation of the immune response to influenza vaccination and should therefore be performed for every new vaccine,^17^ the difficulty is in deciding what to measure, when to measure and how to measure and it is here that the guideline lacks specificity. Here too, a clear correlation with protection has not been established and the interpretation of results will be challenging. As the scientific understanding of the mechanisms through which CMI conveys protection evolves, so will the ability to set clear requirements and to determine what aspects of CMI can best be used to characterise the immune response and bring understanding to the level of protection that vaccines can elicit in different target groups. Until such time, regulators, manufacturers and scientists will need to maintain a dialogue to improve the characterisation of influenza vaccines.

The guideline additionally states that the neuraminidase antibody (NA) response to vaccination should be determined where appropriate. NA has been found to play a role in the prevention of clinical disease, whereas HA inhibits infection and viral replication.^18^, ^19^, ^20^, ^21^, ^22^ As, ultimately, influenza vaccination aims at preventing clinical disease, insight into the NA response for new influenza vaccines could be an important step in achieving a better characterisation of the clinical characteristics of influenza vaccines. However, the amount of NA is not standardised in current influenza vaccines. Therefore, for these vaccines, it does not make sense to determine the NA response, however should be considered in the development of future influenza vaccines.

The challenges regarding assay standardization apply to all these assays mentioned. Certain measures are proposed to minimise the impact, for using using a single centralised laboratory, employing validated assays and international standards where available, and using in‐house controls and unified protocols. Although some of these may prove logistically challenging, the variability in assays necessitates these steps. It would be impossible, for example, to rely on different laboratories to analyses samples from a single study. Ongoing research and collaboration between public health institutes, regulators, manufacturers and academia focussing on the standardisation and development of assays can be expected to result in improved assays and assay reproducibility.^23^

A consequence of abandoning the CHMP criteria is a change to the requirements in the presentation of immunogenicity data. Data from the SRH, HI and VN assays should be presented according to geometric mean titres (GMTs) and reverse cumulative distribution curves (RCDCs). In addition, seroconversion rates should be given. As there is no set definition for seroconversion, several definitions could be applied when presenting the data. GMTs are a summary measure which can be useful in comparing responses between two groups. The RCDCs will allow the visualisation of the immune response across the population. These changes will allow for a more comprehensive assessment of the vaccine‐induced immune response than under the former guideline, which often resulted in the simple conclusion ′The CHMP criteria were met′.

## How to establish clinical efficacy in the post‐CHMP criteria era?

As stated earlier, serological data alone will no longer be sufficient to conclude whether a vaccine is protective in the target population. The new approach for seasonal and for zoonotic and pandemic vaccines is outlined below.

### Seasonal vaccines

For persons over 18 years of age, the proposed guideline states that efficacy of seasonal, non‐adjuvanted inactivated vaccines can be determined in a direct head to head comparison either with a licensed vaccine or with a similar construct for which there is “at least some data to support effectiveness”. If the immune response of the new vaccine is non‐inferior, it is thought reasonable to assume the protective efficacy would at least be comparable.

For children younger than 3 years, there is inconsistent evidence on the efficacy and effectiveness of seasonal inactivated vaccines.^24^, ^25^ Efficacy in this age group cannot be assumed for existing vaccines and cannot therefore be deduced from comparative immunogenicity studies. Hence, the proposed guideline requires applicants to conduct randomized controlled trials with clinical endpoints in order to conclude efficacy for children aged 6 months to 3 years. For children between the ages of 3–6 years, there is some evidence to support efficacy of inactivated influenza vaccines, albeit being moderate.^24^, ^25^, ^26^ Yet the proposed guideline states that as the proportion of children up to the age of approximately 9 years who are immunologically primed is thought to be variable, efficacy can be deduced from demonstrating a non‐inferior immune response to the youngest children for whom efficacy against clinical endpoints should have been demonstrated. For children over the age of nine, the approach taken in the proposed guideline is similar to the approach in adults.

### Zoonotic vaccines and pandemic vaccines

Zoonotic and pandemic vaccines pose a regulatory challenge. Prior to licensure, it is not possible to obtain efficacy data, and the clinical package will be limited to immunogenicity and safety data. Moreover, ethical considerations of testing vaccines in human subjects when there is no direct benefit to the recipient, as there is no immediate threat of a circulating virus, certainly have an impact on regulatory expectations. No firm requirements are set for children, it is merely stated that immunogenicity and safety data in this age group should be obtained “as far as may be possible”.

## Requirements regarding annual changes in seasonal inactivated vaccines

For seasonal influenza vaccines, the annual change in composition has always posed a unique challenge, that is how to determine the impact of the change in viral strains on the clinical characteristics of the vaccine in a short timeframe between production and epidemic. There has been a substantial shift in the proposed guideline. Previously, the CHMP required manufacturers of inactivated influenza vaccines to conduct small clinical trials in 100 adults, including 50 subjects aged ≥60 years, to demonstrate that immunogenicity and reactogenicity were not affected by the strain change.

These trials are not able to detect changes in the clinical characteristics of influenza vaccines.^27^ More importantly however, it is unlikely that a change in vaccine strains as a result of antigenic drift will affect the clinical characteristics of these vaccines to such a degree that the benefit risk balance is radically altered. Consequently, these trials are no longer required. The proposed guideline and an earlier published annex to this guideline^28^ instead move towards closer monitoring of seasonal influenza vaccine performance.

## Moving towards sustainable monitoring of vaccine performance

### Effectiveness

For all seasonal influenza, vaccines licensed in Europe a Risk Management Plan (RMP) will be required which should include the monitoring of influenza vaccine effectiveness (IVE).

From a regulatory perspective, the monitoring of IVE would fit into the lifecycle approach of medicines. It will inform the evolution of the benefit risk balance, allow the detection of potential issues with effectiveness and provide data on the benefits to balance potential safety issues. In addition, once well established, these routine studies could provide a platform to address questions surrounding the performance of new influenza vaccines that are difficult to address pre‐licensure, and to measure product‐specific effectiveness in a pandemic.

Observational studies into IVE are notoriously subject to bias,^29^ and the success of this measure will depend on the robustness of the study protocols and implementation thereof. Moreover, studies should ideally be capable of reporting effectiveness estimates in a timely manner and provide brand‐specific estimates, potentially challenging the feasibility of this exercise.

The proposed guideline builds upon experience already gained in the field through initiatives such as the European I‐MOVE collaboration^30^ and encourages manufacturers to tap into this experience and use existing networks. It refers to protocols developed by the European Centre for Disease Control (ECDC). These include (test negative) case–control studies, cohort studies and screening studies. Influenza cases have to be laboratory confirmed via either RT‐PCR or culture, although within the cohort design, non‐specific endpoints such as medically attended influenza like illness, all‐cause deaths, intensive care admissions and hospitalisations for all respiratory conditions are considered endpoints of interest. When conducting a cohort study, the guideline requires a nested (test negative) case–control study to confirm the effectiveness against laboratory‐confirmed influenza, ensuring a specific measure of effectiveness is available. For details on most aspects, the guideline refers back to the ECDC protocols.

The measurement of IVE is a challenging undertaking, and it should not be the expectation that requested studies will provide clear answers during the first few years. The landscape of vaccination in Europe is diverse, and although this diversity can be an advantage when evaluating vaccines, it will prove a challenge when implementing IVE monitoring. Not only will the epidemiology differ between regions, vaccination policies vary between countries as does the uptake of vaccines and vaccines used. Moreover, vaccination registries are not operational in all countries and regions within the EU.^31^ Where they are in place, it is not always possible to link these to outcome data such as electronic healthcare data. This will certainly limit the initial ability to conduct larger scale studies that could provide product‐specific estimates in selected target groups.

It is important to realise that IVE is not only a consequence of the product used but of a range of determinants such as the vaccination programme and viral epidemiology which play an important role. Any estimates obtained will have to be placed within the context of the myriad determinants of IVE, many of which are poorly understood. This underlines the shared responsibilities between manufacturers, public health institutes and regulators in evaluating and assessing vaccine effectiveness.

### Safety

The monitoring of safety is central to the monitoring of vaccine performance. In Europe, routine pharmacovigilance activities are currently the main source for the identification of potentially serious but rare adverse events following influenza vaccination, and they rely heavily on passive reporting. This comes with limitations as it does not allow for estimation of the incidence of specific adverse events or the association with vaccination. Although the safety of inactivated influenza vaccines that have been used over recent decades is well characterised,^26^, ^32^, ^33^ there is always the possibility of serious adverse events occurring following manufacturing changes, contamination of batches or through the introduction of new pandemic influenza strains. Moreover, the introduction of new influenza vaccines would necessitate intensive surveillance of their safety as clinical trials are insufficient to detect rare but serious adverse events.

Whilst some European countries have the infrastructure in place to rapidly evaluate safety signals, this capacity is fragmented. Moreover, countries are often too small, or vaccine use is too limited, to properly evaluate rare safety signals. As mentioned earlier, vaccination registries do not exist in all countries and regions of Europe, and it is not always possible to link vaccination data to outcome data.^31^ With an increasing need for rapid evaluation of safety signals in order to provide timely guidance to policymakers and address public concerns, there is a clear need to invest further in European systems to monitor and evaluate the safety of vaccines.

The proposed guideline requires that the RMP includes plans for enhanced surveillance of vaccine safety, as detailed in an Annex to the guideline.^28^ The aim of this enhanced surveillance is to rapidly detect a significant increase in reactogenicity that would signal potential serious risks following annual strain changes. Adverse events of interest include typical local and systemic reactions to vaccination such as rash, injection site reactions, myalgia, fever, nausea and headache. To achieve this, defined cohorts of children and adults, including a minimum total of 500 persons–100 per age stratum, should be followed after vaccination for the occurrence of several adverse events of interest. Rates of adverse events will have to be compared to rates in previous years. Alternatively, enhanced passive surveillance could be employed in which the reporting of adverse events is facilitated to obtain reporting rates which can function as a surrogate for the adverse events of interest. Furthermore, data mining of electronic health record data can be also employed. However, such mining has the clear limitation of the near impossibility of gathering information on vaccine reactogenicity from electronic healthcare databases.

Although the increased attention to the monitoring of influenza vaccine safety is welcomed, it is questionable whether the proposed enhanced surveillance is the most efficient means to achieve the goal; the rapid identification of safety signals has the ability to thoroughly evaluate the association between the signal and vaccination. It would seem more sensible to further invest in the creation of vaccine registries in Europe, improve the registration of vaccination data in existing registries, facilitate the linkage of these registries to electronic healthcare databases, limit the data lag for registries and databases and invest in the capacity to implement rapid signal detection and evaluation. Such an infrastructure would permit continuous monitoring and evaluating the safety of influenza vaccines, also after annual strain changes. It is unlikely that data on vaccine reactogenicity in 500 persons will be predictive of any serious but rare adverse events and whether the studies will be able to discriminate relevant changes from year to year that could predict adverse events which could alter the BR balance of the vaccines.

The 2014/2015 influenza season was the first season for which the enhanced surveillance should have been up and running, and time will tell how suitable these studies are in detecting potential safety signals associated with the updating of influenza strains in seasonal vaccines.

## Final considerations

Lessons learned during the influenza A(H1N1)2009 pandemic together with advances in the scientific understanding of influenza and the immune response to influenza viruses and vaccines have resulted in the revision of existing regulatory guidelines for the licensing of influenza vaccines in Europe. Following a public consultation round, it is expected that the final guidance will be published in the near future.

The proposed guideline reflects a changed approach to the regulatory assessment of influenza vaccines. This has resulted in the abolition of the CHMP criteria, the introduction of more diversified requirements for measuring and reporting the immune response to influenza vaccines, and the requirement for all new influenza vaccines to conduct trials with clinical outcomes in children aged 6–36 months. Furthermore, immunogenicity data are no longer requested to support annual strain changes. Importantly, more emphasis is placed on the post‐licensure monitoring of the benefit risk of influenza vaccines, including a request for continuous monitoring of efficacy and enhanced safety surveillance.

Presently, several gaps remain in the understanding of the performance of seasonal influenza vaccines. It is expected that the changes made to the influenza guideline will improve the characterisation of clinical characteristics of new and existing influenza vaccines. The new requirements will certainly improve our knowledge on the functioning of influenza vaccines in children and can be expected to provide a better insight into the immune response overall. The move towards sustained monitoring of the benefits and risks of influenza vaccines underlines regulation does not stop at licensure, and will undoubtedly lead to more accurate data on the benefits and risks to address public concerns should these arise.

Major challenges, however, remain, such as the absence of standardised serological assays and the absence of a correlate of protection to facilitate vaccine evaluation. Moreover, the limited availability of an infrastructure in Europe which would allow timely and consistent evaluation of the effectiveness and safety of vaccines currently impedes adequate benefit risk monitoring of new influenza vaccines. New guidance cannot overcome these challenges, and regulators can merely encourage investment in improved methods.

Manufacturers are responsible for their products, regulators guard those products, and public health institutes are responsible for the programmes in which the vaccines are used. Improving the evaluation of vaccines is therefore a shared responsibility between manufacturers, regulators and public health institutes – all of which are dependent on academia for scientific input. This recognition, in addition to the identified need for improved methods and collaboration for vaccine evaluation, has resulted in EU‐wide collaboration between public health institutes, industry, regulators and academia which aims to improve the benefit risk monitoring of vaccines^34^ and serological assays for evaluating influenza vaccines.^23^ Collaborative initiatives like these will ultimately result in improved vaccines a better understanding of their immunology and clinical performance, but also more robust tools to monitor performance of influenza vaccines in the future.
